# Changes in ideal cardiovascular health among Iranian adolescents: 2007–2008 to 2015–2017

**DOI:** 10.1186/s12887-022-03504-x

**Published:** 2022-07-26

**Authors:** Golaleh Asghari, Parvin Mirmiran, Alireza Rezaeemanesh, Maryam Mahdavi, Fereiodoun Azizi, Farzad Hadaegh

**Affiliations:** 1 Nutrition and Endocrine Research Center, Research Institute for Endocrine Sciences, Shahid Beheshti University of Medical Sciences, Tehran, Iran; 2 Department of Clinical Nutrition and Dietetics, Faculty of Nutrition Sciences and Food Technology, National Nutrition and Food Technology Research Institute, Shahid Beheshti University of Medical Sciences, Tehran, Iran; 3 Prevention of Metabolic Disorders Research Center, Research Institute for Endocrine Sciences, Shahid Beheshti University of Medical Sciences, Tehran, Iran; 4 Obesity Research Center, Research Institute for Endocrine Sciences, Shahid Beheshti University of Medical Sciences, Tehran, Iran; 5 Endocrine Research Center, Research Institute for Endocrine Sciences, Shahid Beheshti University of Medical Sciences, Tehran, Iran

**Keywords:** Cardiovascular, American Heart Association, Adolescent

## Abstract

**Background:**

Assessment of both behavior and factors of health as ideal cardiovascular health (iCVH) in adolescence could contribute to cardiovascular disease prevention in adulthood.

**Aims:**

To explore the changes in the prevalence of iCVH and its components during a decade among Tehranian adolescents.

**Methods:**

The 12–19 years old adolescents were selected from the Tehran Lipid and Glucose Study(TLGS). The iCVH score was calculated in the study period 1 (2007–2008; *n* = 267) and 2 (2015–2017; *n* = 336). To calculate iCVH, body mass index(BMI), systolic and diastolic blood pressure (BP), total cholesterol, fasting plasma glucose (FPG), physical activity, smoking status, and dietary intakes were measured by standard protocols. The changes in iCVH components between the two study periods were reported by prevalence (95% confidence interval). A logistic regression model was conducted to test the effects of study periods, sex, and age groups on the iCVH(≥ 6 scores).

**Results:**

Overall, there was a reduction in the prevalence of ideal FPG (97.4 vs. 91.1%) and ideal BP(91.8 vs. 82.7%). Girls had a decrease in the prevalence of ideal BP(91.2 vs. 79.4%) as well as an increase in non-smoking status(77.6 vs. 89.7%). However, the prevalence of ideal FPG (96.5 vs. 88.5%) and ideal BP(92.2 vs 85.0%) decreased in boys. Study period 2, compared to period 1 was associated with lower odds of having iCVH. Furthermore, boys were 1.57 folds more likely to have ideal CVH factors than girls.

**Conclusion:**

There was a decrease in the prevalence of ideal CVD metrics, including FPG and BP, after one decade. Generally, adolescent boys had higher odds of having ideal CVH compared to girls.

**Supplementary Information:**

The online version contains supplementary material available at 10.1186/s12887-022-03504-x.

## Introduction

Cardiovascular disease (CVD) is the leading cause of morbidity and mortality worldwide [1]. Moreover, the high incidence of premature CVD and mortality events is an important problem in the Middle East and North Africa (MENA) region [2]. As cardiovascular health in early life is highly correlated with CVD status in adulthood, assessment of CVD risk factors becomes an important task in childhood and adolescence [3]. There has been a rising trend in unhealthy lifestyles as well as CVD risk factors among adolescents residing in the MENA region, leading to premature CVD events [4–7].

In 2010, The American Heart Association established a construct “ideal cardiovascular health (iCVH)” to measure cardiovascular health [8]. This concept includes four ideal behaviors (no smoking, physical activity, a normal body mass index (BMI), and a healthy diet) as well as three ideal health factors (normal glucose levels, blood pressure, and serum total cholesterol). By using this method, researchers were able to monitor cardiovascular health in the general population and measure the achievement of AHA goals [8].

A large population-based cohort study in the US among adults has reported that adherence to healthy lifestyle practices can be effective in the prevention of the majority of cardiovascular events [9, 10]; also, another study has shown that the number of iCVH metrics is a key predictor of CVD and its mortality [11]. Furthermore, several studies have shown that having a higher number of ideal cardiovascular health behaviors and factors is associated with improving liver biomarker levels [12], a lower inflammatory profile [13], and a lower risk of premature CVD and mortality [10].

During the last decade, several studies have been conducted among children and adolescents in different populations to examine the status of iCVH [14–16]. The 2005–2010 National Health and Nutrition Examination Surveys reported that less than 50% of American adolescents had ≥ 5 iCVH components [14]. Another study conducted among the European adolescents was also found revealed a low prevalence of iCVH behaviors, particularly diet and physical activity [15]. In addition, in the National Growth and Health Study (NGHS), the ideal cardiovascular health status among girls in the United States declined from 30% in early adolescence to 10% in early adulthood [16]. Among Chinese adolescents, an adverse trend in iCVH from 2004 to 2014 was observed, particularly among girls and younger age groups. [17]

Although several studies in the MENA region have examined the prevalence of specific CVD risk factors and metabolic syndrome among adolescents [18–20], there is no information on the overall trend of iCVH among adolescents. Thus, in this study, we examined the changes in the prevalence of iCVH and its components during a decade in a cohort of 12–19 year old Tehranian adolescents.

## Methods

### Study participants

Data were collected within the framework of the Tehran Lipid and Glucose Study (TLGS), a community-based ongoing prospective cohort designed to investigate the risk factors of non-communicable diseases and to promote healthy lifestyle in District 13 of Tehran, Iran, with follow-up examinations every 3 years, starting from 1999. Additional information about the TLGS design and profile can be found elsewhere [21]. The study examination cycles were as follows: I (1999–2001), II (2002–2005), III (2006–2008), IV (2009–2011), V (2012–2015), and VI (2015–2017). For the current study, to examine the changes in the prevalence of iCVH, we conducted the study in two time periods of 2006–2008 (III) and 2015–2017 (V). The study population of 12,523 participants, of whom 3462 were randomly selected for dietary assessment in 2007–2008 (the characteristics of participants who completed the dietary assessment were similar to those of the total population [22]). From 3462, 446 were aged between 12 to 19 years. After excluding those with missing data on iCVH components, 267 adolescents remained for final analysis in study period 1 (2007–2008). Accordingly, a total number of 11,415 participants, of whom 7721 were randomly selected for dietary assessment in 2015–2017. From 7721, 569 were aged 12–19 years. After excluding those with missing data on iCVH components, 336 adolescents remained for final analysis in study period 2 (2015–2017). There were no common individuals among the population selected from the two time period.

### Measurement

Data on smoking status were collected using standard questioners. Physical activity was assessed using Modifiable Activity Questionnaire (MAQ) expressed as metabolic equivalent minute per week (MET-min/week) were obtained [23]. Anthropometric measurements were carried out with light clothing and without shoes. Weight and height were measured by a digital scale and an elastic tape in the standard position, respectively. Waist circumference was recorded at the umbilicus level. At follow-ups, standardized protocols were utilized as previous descriptions [21]. The body mass index (BMI) was calculated by dividing weight in kilograms by the height in meters squared.

After 15 min of resting, the systolic and diastolic blood pressure (SBP, DBP) of the participants were measured using a mercury sphygmomanometer by an experienced physician. additionally, a blood sample was drawn between 7:00 to 9:00 am into vacutainer tubes after 12–14 h of overnight fasting to measure total cholesterol (TC) and fasting plasma glucose (FPG). In addition, the dietary assessment was conducted by face-to-face interviews by trained dietitians using a valid and reliable FFQ related to the past 12 months [24].

### Definition of ideal cardiovascular health

According to AHA [8], iCVH consists of two components: the first component is health behaviors which is based on non-smoking status, ideal BMI, favorable physical activity, and a healthy dietary pattern; the second component is favorable health factors consisting of blood pressure, total cholesterol, and FPG. In detail, the ideal status of smoking was defined as never trying a cigarette smoking or hookah. The ideal BMI was defined as < 85th percentiles of BMI for age and sex based on the standardized percentile curves of BMI suggested for Iranian children and adolescents [25]. The ideal status of physical activity was defined as > 600 MET/min/week. The components of a healthy dietary pattern were defined as consuming fruits and vegetables ≥ 4.5 cups/d, fish two 3.5-oz servings/w, whole grains ≥ three 1-oz-equivalent servings/d, sodium < 1500 mg/d, and sugar-sweetened beverages ≤ 450 kcal/w. The portion sizes have been considered in a 2000-kcal diet, and we modified them according to other levels of caloric intake. The participants were considered to have an ideal status of the dietary pattern if they had ≥ 4 of the above components appropriately. The ideal status of blood pressure was defined as systolic and/or diastolic blood pressure < 90th percentile for sex, age, and height according to the Heart, Lung, and Blood Institute standards [26]. Ideal serum cholesterol and FPG were defined as total cholesterol < 170 mg/dl and FPG < 100 mg/dl, respectively. BMI Z-score was defined according to age- and sex-specific World Health Organization criteria [27].

### Statistics

Participant characteristics will be summarized by a number of ideal CVH variables. To ensure the presence of sufficient numbers within each group, we will categorize participants into those with ideal and poor CVH variables.

Normally distributed and skewed continuous variables are illustrated as Mean ± SD and median (IQR 25–75), respectively. We used Student's t-test, Mann–Whitney test, or Chi-square to compare the study variables as well as the prevalence of ideal cardiovascular health metrics among adolescents in study period 1 (2007–2008) and study period 2 (2015–2017). A logistic regression model was utilized to investigate the association of study period, sex, and age groups with ideal CVH (≥ 6 scores). All analyses were performed using STATA version 14 SE (STATA Inc., TX, USA), with a two-tailed *P*-value, 0.05 being considered significant.

## Results

The mean age of study participants during both study periods were 15.5 years, with 37.5% in the 12–14 year old age group and 62.5% in the 15–19 year old age group. Characteristics of boys and girls during 2007–2008 and 2015–2017 are reported in Table 1. Among girls, the mean waist circumference, FPG, DBP, BMI z-score, and BMI were significantly higher after one decade.

**Table 1:** Characteristics of adolescents in TLGS phase III (2007–2008) and phase VI (2015–2017)

	Boys		***P*** **-value**	Girls		***P*** **-value**
	**Phase III (2007–2008)**	**Phase VI (2015–2017)**		**Phase III (2007–2008)**	**Phase VI (2015–2017)**	
Age (years)	15.1 ± 2.1	15.4 ± 2.2	0.326	15.9 ± 2.4	15.6 ± 2.4	0.310
Age categories			0.345			0.300
12–14 year old (%)	59 (41.5)	73 (36.5)		41 (32.8)	53 (39.0)	
15–19 year old (%)	83 (58.5)	127 (63.5)		84 (67.2)	83 (61.0)	
Waist (cm)	81.3 ± 13.4	80.4 ± 12.3	0.535	71.8 ± 9.1	79.6 ± 10.4	** < 0.001**
Fasting plasma glucose (mg/dl)	85.3 ± 13.2	90.3 ± 10.4	** < 0.001**	83.1 ± 10.9	86.5 ± 15.2	**0.044**
Systolic blood pressure (mmHg)	104.8 ± 11.7	105.1 ± 13.8	0.847	99.6 ± 11.7	101.3 ± 14.4	0.304
Diastolic blood pressure (mmHg)	67.5 ± 9.1	70.3 ± 10.6	**0.013**	66.8 ± 8.6	70.4 ± 11.7	**0.006**
Cholesterol (mg/dl)	146.7 ± 34.6	149.8 ± 33.1	0.407	153.8 ± 30.6	147.0 ± 30.6	0.073
Body mass index z-score	0.97 (-0.3, 2)	1.02 (-0.84, 2)	0.604	0.39 (-0.37,1.6)	0.82 (-0.11,2.08)	**0.058**
Body mass index (kg/m^2^)	22.8 ± 5.1	22.6 ± 4.4	0.623	22.4 ± 4.2	23.6 ± 4.8	**0.031**
Physical activity (MET/h/wk)	1124 (536, 2166)	1033 (397, 1901)	0.303	402 (186, 949)	357 (123, 796)	0.187
Dietary components						
Fruits and vegetables	59 (41.5)	73 (36.5)	0.345	68 (54.4)	63 (46.3)	0.192
Fish	11 (7.7)	7 (3.5)	0.083	7 (5.6)	5 (3.7)	0.459
Whole grain	5 (3.5)	40 (20.0)	** < 0.001**	4 (3.2)	19 (14.0)	**0.002**
Sodium	8 (5.6)	4 (2.0)	0.082	7 (5.6)	10 (7.4)	0.566
Sweetened beverages	134 (94.4)	176 (88.0)	**0.046**	122 (97.6)	130 (95.6)	0.504

However, boys in the 2015–2017 period had higher FPG and DBP than in 2007–2008 period. Dietary factors were not significantly different between the two groups except for whole grain in both sexes and sugar-sweetened beverages among boys (*P* < 0.05).

As shown in Table 2, the prevalence of ideal FPG and blood pressure were significantly lower in adolescents of the 2015–2017 period compared to those from 2007–2008 (97.4 vs 91.1% and 91.8 vs 82.7%, respectively). In both study periods, only one participant had ideal dietary status. The mean (95% CI) of iCVH metrics was 4.6 (4.5–4.8) in 2007–2008 and 4.5 (4.4–4.6) in 2015–2017 (*P* = 0.052). The prevalence of those with ≥ 6 ideal components of CVH metrics were 22.5% (17.8–27.9) in 2007–2008 and 16.7% (13.0–21.0) in 2015–2017.

**Table 2:** Prevalence of ideal cardiovascular health metrics in adolescents in study period 1 (2007–2008) and study period 2 (2015–2017)

	Phase III (2007–2008)		Phase VI (2015–2017)		*P*-value
	No	Prevalence (95% CI)	No	Prevalence (95% CI)	
Smoking status	199	74.5 (68.9–79.4)	265	78.9 (74.1–82.9)	0.209
Physical activity	150	56.2 (50.1–62.0)	180	53.6 (48.2–58.9)	0.371
Body mass index	169	63.3 (57.3–68.9)	198	58.9 (53.6–64.1)	0.275
Healthy diet score	1	0.37 (0.05–2.66)	1	0.3 (0.04–2.1)	0.870
Total cholesterol	216	80.9 (75.7–85.2)	277	82.4 (78.0–86.2)	0.626
Fasting plasma glucose	260	97.4 (94.6–98.7)	306	91.1 (87.5–93.7)	0.001
Blood pressure	245	91.8 (87.8–94.5)	278	82.7 (78.3–86.4)	0.001
No. of ideal CVH metrics					
0	-	-	-	-	
1	-	-	-	-	
2	9	3.4 (1.7–6.4)	9	2.7 (1.4–5.1)	0.620
3	27	10.1 (7.0–14.4)	48	14.3 (10.9–18.5)	0.123
4	75	28.1 (23.0–33.8)	109	32.4 (27.6–37.6)	0.249
5	96	35.9 (30.4–41.9)	114	33.9 (29.0–39.2)	0.604
≥ 6	60	22.5 (17.8–27.9)	56	16.7 (13.0–21.0)	0.072
Mean CVH metrics (95% CI)		4.6 (4.5–4.8)	4.5 (4.4–4.6)		0.052

*TLGS* Tehran Lipid and Glucose Study, *CI* confidence interval, *CVH metrics* cardiovascular health index

Among girls, the prevalence of ideal blood pressure was significantly lower (91.2 vs. 79.4%); however, the prevalence of non-smoking status was higher in 2015–2017 (77.6 vs. 89.7%, *P* = 0.008). None of the participants had an ideal dietary component in either study period. The mean (95% CI) of iCVH metrics among girls was 4.5 (4.3–4.7) in 2007–2008 and 4.4 (4.2–4.6) in 2015–2017 (*P* = 0.432). Among boys, the prevalence of ideal FPG and blood pressure were significantly lower (96.5 vs 88.5% for ideal FPG and (92.2 vs. 85.0% for ideal blood pressure; *P* < 0.05). The mean (95% CI) of iCVH metrics was 4.8 (4.6–4.9) in 2007–2008 and 4.5 (4.4–4.7) in 2015–2017 among boys (*P* = 0.038). When stratified by age groups, those in the 12–14 and 15–19 year age groups had a lower prevalence of ideal FPG and blood pressure status (Supplementary Fig. 1).

**Fig. 1:**
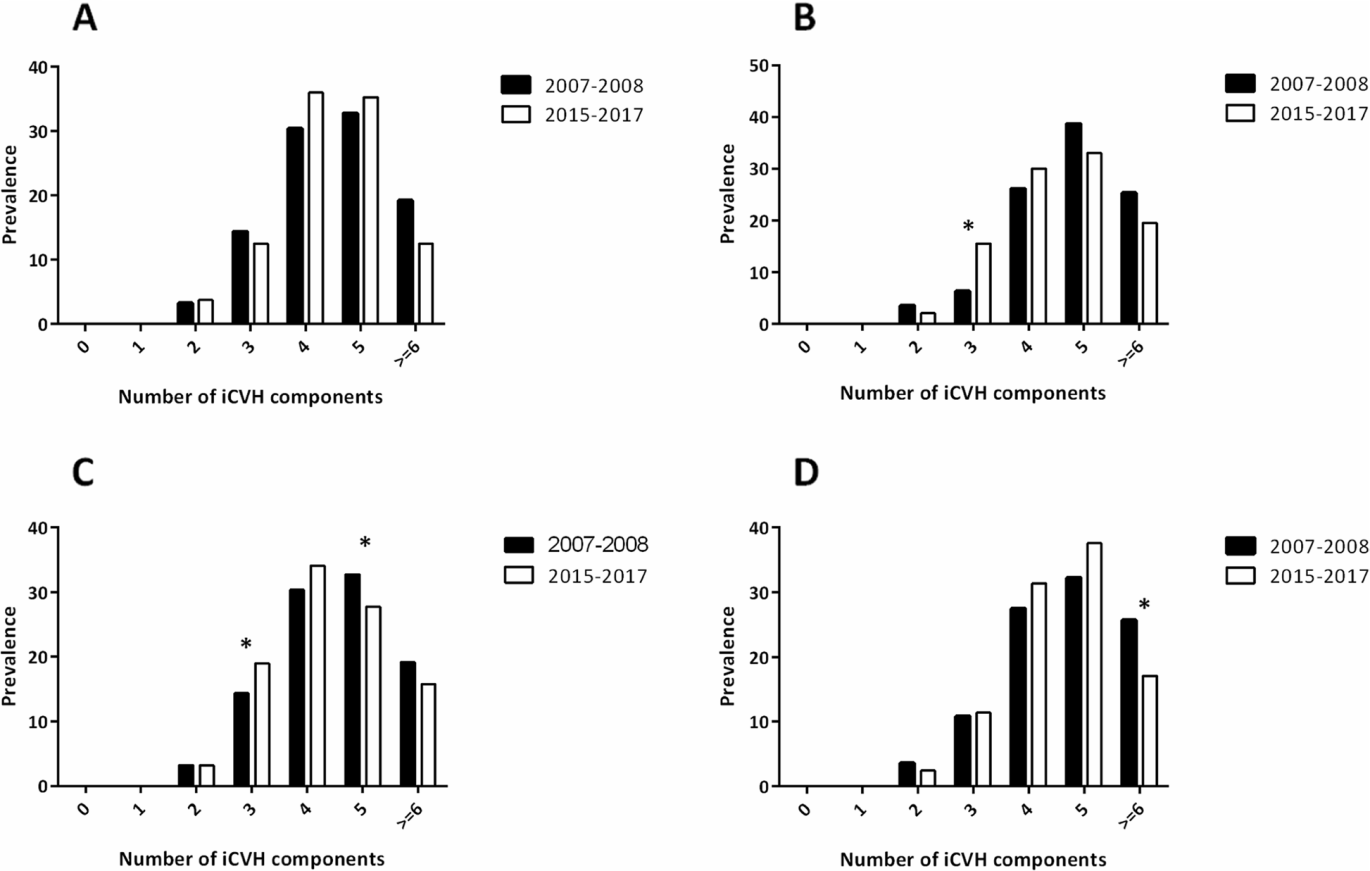
Prevalence of the number of ideal cardiovascular health metrics in adolescents in study period 1 (2007–2008) and study period 2 (2015–2017) stratified by sex and age groups. A: girls; B: Boys; C: 12–14 year age group; D: 15–19 years age group. *P < 0.05

As shown in Fig. 1, among girls, the prevalence of those with ≥ 6 ideal components of CVH metrics was 19.2% (13.2–27.1) in 2007–2008 and 12.5% (7.9–19.2) in 2015–2017. Boys with ≥ 6 ideal components of CVH metrics had a prevalence of 25.3% (18.8–33.2) in 2007–2008 and 19.5% (14.5–25.6) in 2015–2017. Those in the 15–19 age group had a larger reduction between the two study periods in the prevalence of participants with ≥ 6 components (25.7 vs 17.1%) compared to those in the 12–14 year age group (17.0 vs 15.8%).

In the multivariate logistic regression analysis among pooled sample of adolescents, study period 2 compared to study period 1 was associated with lower odds of having an ideal CVH (OR [95%CI]: 0.67 [0.44–1.00]). Furthermore, boys were 1.57 folds (95%CI: 1.03–2.34) more likely to have ideal CVH factors than girls (Table 3).

**Table 3:** Association of the study period, gender, and age group with odds of having ideal cardiovascular health score ≥ 6

	OR	95% confidence interval	*p*-value
**Study period**			
2007–2008	1.00	-	0.052
2015–2017	0.67	0.44–1.00	
**Gender**			
Girls	1.00	-	
Boys	1.57	1.03–2.34	0.037
**Age group**			
12–14 years	1.00	-	
15–19 years	0.72	0.47–1.08	0.115

## Discussion

We examined the prevalence of CVH metrics among Tehranian adolescents aged 12–19 years for two time periods within a ten-year span. Accordingly, there was an almost 6% decrease (from 22.5% to 16.7%) in the prevalence of ideal CVD metrics (i.e. ≥ 6 scores) among the adolescents \ over a decade. Specifically, among different components of CVD health metrics, the trends of ideal FPG (among boys) and BP (in both genders) were significantly lower. Moreover, we also found that adolescent boys generally had 50% higher odds of having an ideal CVH compared to girls.

Due to the use of different cut-offs to define iCVH status (≥ 5, ≥ 6, or 7 ideal components), it is not straightforward to compare our findings with previous studies. An overview of the results of the studies in different populations (from the United States, European countries, China, and Australia) is provided in Table 4. The total prevalence of iCVH among adolescents in different countries ranges from 10% to nearly 50% [14, 15, 17, 28–32]. To the best of our knowledge, no study so far has reported the iCVH prevalence among adolescents in the MENA region, and only two studies (from the U.S and China) have reported the trend of iCVH prevalence [17, 30]. Among Chinese aged 12–18 years, ideal levels of almost all the seven metrics decreased, except for a marked increase in physical activity. Overall, only 19.5% of boys and 22.0% of girls had ideal cardiovascular health (score ≥ 6) in 2004, which worsened in 2014 (boys: 9.8%; girls: 16.0%) [17]. Similar to our study, the ideal status of FPG dropped significantly in both sexes, and the ideal status of BP reduced significantly among boys during one decade [17]. However, the findings from a series of National Health and Nutrition Examination Survey (NHANES) show that the U.S adolescents had a consistent trend of iCVH from 1988 through 2010; with an increase in the prevalence of healthy dietary pattern and non-smoking status, while the prevalence of normal BMI and physical activity got lower, resulting in an overall unchanged iCVH metrics scores between 1988 to 2010 [30]. It seems that the results of the previous studies are not completely consistent, but in general, it can be stated that the prevalence of iCVH metrics in adolescents is low, which can make adolescents prone to the risk of chronic diseases such as metabolic syndrome, hypertension, and atherosclerotic CVD in early adulthood [28].

**Table 4:** Prevalence of ideal cardiovascular health (iCVH) metrics among other studies

Author	Year of study	Country	Population, age range	Smoking (%)	Physical activity (%)	Body mass index (%)	Diet (%)	Cholesterol (%)	Fasting plasma glucose (%)	Hypertension (%)	Total iCVH (%)
Laitinen et al., 2012 [28]	1986	Finland	Young Finns, 12–18 years	T: 22.4	T: 6.9	T: 85.6	T: 24.3	T: 33.2	T: 97.4	T: 82.2	T: -
Yang et al., 2014 [30]	1988–1994 1999–2004 2005–2010	United States	NHANES, 12–17 years	T: 82.8 T: 86.1 T: 88.7	T: 90.4 T: 88.5 T: 86.6	T: 72.4 T: 67.3 T: 65.6	T: 8.4 T: 10.0 T: 11.3	T: 66.5 T: 65.2 T: 70.9	T: 96.4 T: 97.3 T: 95.2	T: 84.9 T: 84.3 T: 85.3	T: 35.5 T: 35.7 T: 37.2
Shay et al., 2013 [14]	2005–2010	United States	NHANES, 12–19 years	G: 70 B: 66	G: 43.5 B: 67	G: 67 B: 66	G: 0 B: 0	G: 65 B: 72	G: 89 B: 74	G: 90 B: 77.7	G: 45.3 B: 49.5
Dong et al., 2016 [17]	2004 2014	China	BCAMS and CCACH, 6–18 years	G: 97.9 B: 97.0 G: 95.5 B: 87.8	G: 16.2 B: 23.0 G: 41.0 B: 52.8	G: 18.2 B: 19.5 G: 18.6 B: 20.2	G: ^*^ B:^*^ G:^*^ B:^*^	G: 4.0 B: 4.0 G: 4.3 B: 4.2	G: 4.6 B: 4.7 G: 5.4 B: 5.6	G: ^†^ B: ^†^ G: ^†^ B: ^†^	G: 22.0 B: 19.5 G: 16.0 B: 9.8
Henriksson et al., 2017 [15]	2006–2007	European countries	HELENA, 12.5–17.5 years	G: 60.3 B: 61.5	G: 56.2 B: 68.6	G: 80.2 B: 73.2	G: 3.0 B: 0.3	G: 55.1 B: 77.8	G: 93.7 B: 83.3	G: 74.3 B: 48.4	G: B:
Liu et al., 2018 [31]	2015–2016	Australia	LSAC, 10–12 years	T: -	T: 25	T: 38	T: 18	T: 69	T: 88	T: 55	T: 39
Agostinis-Sobrinho et al., 2018 [32]	2011	Portugal	LabMed physical Activity, 12–18 years	T: 93.4 G: 95.1 B: 91.2	T: 36 G: 25.1 B: 49.3	T: 87.6 G: 87.4 B: 87.8	T: 47.7 G: 49.2 B: 45.9	T: 76.4 G: 72.7 B: 81.1	T: 85.2 G: 95.1 B: 95.3	T: 90.9 G: 91.2 B: 90.7	T: - G: - B: -
Yan et al., 2019 [29]	2013–2015	China	CCACH, 6–18 years	T: 90.7 G: 95.4 B: 86.3	T: 16.6 G: 12.2 B: 20.7	T: 77.3 G: 84.0 B: 71.3	T: 8.7 G: 8.3 B: 9.1	T: 80.7 G: 78.6 B: 82.7	T: 76.5 G: 80.2 B: 73.1	T: 69.3 G: 77.8 B: 61.3	T: - G: 49.3 B: 35.8
**Current Study**	**2007–2008** **2015–2017**	**Iran**	**TLGS, 12–19 years**	**G: 77.6** **B: 71.8** **G: 89.7** **B: 71.5**	**G: 38.4** **B: 71.8** **G: 33.1** **B: 67.5**	**G: 67.2** **B: 59.9** **G: 57.3** **B: 60.0**	**G: 0** **B: 0.7** **G: 0** **B: 0.5**	**G: 77.6** **B: 83.8** **G: 86.0** **B: 80.0**	**G: 98.4** **B: 96.5** **G: 94.8** **B: 88.5**	**G: 91.2** **B: 92.2** **G: 79.4** **B: 85.0**	**G: 19.2** **B: 25.3** **G: 12.5** **B: 19.5**

*NHANES* National Health and Nutrition Examination Surveys, *BCAMS* Beijing Child and Adolescent Metabolic Syndrome, *CCACH* China Child and Adolescent Cardiovascular Health, *HELENA* Healthy Lifestyle in Europe by Nutrition in Adolescence study, *LSAC* Longitudinal Study of Australian Children, *LabMed* Longitudinal Analysis of Biomarkers and Environmental Determinants, *TLGS* Tehran Lipid and Glucose Study, *T* total population, *G* girls, *B* boys

^*^ Fruits and vegetables: 2004 (G: 72.2, B: 62.4) and 2014 (G: 67.1, B: 63.7)

Soybean products: 2004 (G: 6.0, B: 7.7) and 2014 (G: 17.4, B: 17.6)

Fish or fish products: 2004 (G: 61.9, B: 60.6) and 2014 (G: 36.9, B: 31.7)

Sugar-sweetened beverage: 2004 (G: 42.3, B: 37.9) and 2014 (G: 36.6, B: 38.8)

Salty snacks: 2004 (G: 87.2, B: 89.2) and 2014 (G: 87.6, B: 88.8)

^†^ Systolic blood pressure (mmHg): 2004 (G: 99.5, B: 104.4) and 2014 (G: 105.1, B: 110.2)

Diastolic blood pressure (mmHg): 2004 (G: 63.8, B: 65.7) and 2014 (G: 62.3, B: 62.4)

Our data analysis showed that adolescent girls generally had more unfavorable CVH metrics. Importantly, girls had increasing values of BMI and WC compared to boys who did not show such findings. WC mainly manifests abdominal fat [33]. These results might be explained by the mechanism that existed between obesity and its influence on vascular function which ultimately leads to BP. It has been proved that the changes in cardiac output and renin–angiotensin–aldosterone system activation consequence of excess weight can lead to high blood pressure [34, 35]. The changes in Iranian socio-economic status, having lower physical activity and higher CVD risk factors in girls could give rise to the following unhealthy behaviors and factors accounted for iCVH. Recent studies have suggested strategies that improve CVH components among adolescents. It has been consistently established that healthy eating and regular physical activity can decrease the risk of developing CVD in children and adolescents. Continuing efforts are needed to promote healthy eating and physical activity among adolescents at the national, local, and school levels, including the implementation of the School Health Guidelines to Promote Healthy Eating and Physical Activity [30]. Considering the importance of eating habits in addressing obesity problems in adolescents, it has been demonstrated that factors such as adequate dietary fiber consumption and eating breakfast regularly can be associated with lower occurrence of overweight/obesity in adolescents [36].

In the current study, in adolescent boys, the prevalence of ideal BP and FPG was significantly lower after one decade, whereas, no significant difference was observed in the prevalence of ideal BMI and waist circumference. Our findings suggest that probably other (unmeasured) factors and not obesity, can explain the lower prevalence of ideal blood glucose and blood pressure levels after one decade in boys. Dietary inflammatory index [37] and lifestyle inflammatory score [38] are associated with increased risk of pre-diabetes, diabetes, and metabolic disorders, independent of possible confounding factors such as energy intake and BMI. Also, the insulinemic potential of diet and lifestyle [39, 40], independent of potential confounders, is associated with a higher risk of type 2 diabetes and insulin resistance in the Iranian population. Furthermore, environmental factors such as endocrine disrupting chemicals may also play a role in the risk of endocrine-related disorders among Iranian children as a more vulnerable population. The exposure to environmental pollution and heavy metals can be suggested as a main potential contributor to increasing the risk of glucose intolerance among Iranian youth with possible mechanisms such as an increased risk of β-cell failure and insulin resistance [41–43]. Importantly, in recent years, a higher burden of air pollution has been reported in Iran, especially in the metropolitan of Tehran [44]. Moreover, our results show that study period 2 (2015–2017) compared with study period 1 (2007–2008) was associated with a lower odds of having ideal CVH status (≥ 6 scores). Another factor which can contribute to higher BP and FPG among boys after one decade is higher consumption of Sugar-sweetened beverages (SSB) in the later period (Table 1). Previously it has been shown that there is an association between SSB consumption and hypertension in Iranian adolescents [45]. Along with our findings, a dose–response meta-analysis demonstrated that the children and adolescents who were SSB consumers were more likely to have hypertension [46]. Moreover, consistent with our results, children who consume higher amounts of SSB are more likely to have insulin resistance and increased blood glucose [47, 48]**.** As one of the SSB ingredients, fructose can increase BP by increasing the reabsorption of salt and water in the small intestine and kidney [49]. Likewise, hyperglycemia that is caused by consuming SSB is associated with weight gain and insulin resistance [50].

The present study has several strengths. To the best of our knowledge, this is the first study in the MENA region to assess the changes in iCVH based on two separate cross-sectional surveys among adolescents during a decade. Also, we have performed a stratified analysis to report the changes in iCVH based on sex and age groups (early and late adolescence). It is important to point out that there are some limitations as well. We had no data on iCVH metrics such as smoking, diet, BMI, blood glucose status, and blood pressure from the participant’s family (parents), which could be helpful in assessing the potential role of genetic background. Furthermore, although major confounding variables were considered in our analyses, there may still be residual or unmeasured confounders such as inflammatory indices and puberty stage. Also, because of the observational nature of our study, it was not possible to conclude causality for the changes in each of the CVD health metrics. Finally, this study was conducted among adolescent residents of Tehran, so it might not be possible to generalize our findings to other parts of the country, especially the rural zones.

In conclusion, cardiovascular health worsened among adolescents in Tehran during one decade from 2007–2008 to 2015–2017. This trend was more prominent regarding blood glucose and blood pressure. Other components of iCVH generally remained the same between the two study periods. Furthermore, boys had a higher odds of having ideal CVH in comparison to girls. These findings call for effective intervention programs to promote healthy lifestyles among Iranian adolescents with a more specific focus on girls. It is suggested that future studies can explore the predictive factors of the changes in ICVH.

## Supplementary Information

**Additional file 1:** 

## Data Availability

The datasets used and/or analysed during the current study available from the corresponding author on reasonable request.
