# Supplementary material for: Changes in ideal cardiovascular health among Iranian adolescents: 2007–2008 to 2015–2017
Source: BMC Pediatr. 2022 Jul 26;22:450. doi: 10.1186/s12887-022-03504-x (PMC9316362; doi:10.1186/s12887-022-03504-x)
Supplement: Supplementary file 1 — Additional file 1: [file 12887_2022_3504_MOESM1_ESM.docx]

**Legend to Supplementary Figure 1**

Prevalence (95% confidence interval) of ideal cardiovascular health metrics in adolescents in study period 1 (2007-2008) and study period 2 (2015-2017) stratified by sex and age groups. A: girls; B: Boys; C: 12-14 year age group; D: 15-19 years age group. ^*^*P*<0.05
